# Vagus Nerve Stimulation Therapy for Epilepsy: Mechanisms of Action and Therapeutic Approaches

**DOI:** 10.3390/brainsci15111236

**Published:** 2025-11-18

**Authors:** Klesta Cocoli, Justine Curley, Pratik Rohatgi, Myriam Abdennadher

**Affiliations:** 1 Boston Medical Center, Boston, MA 02118, USA; 2 LivaNova, Houston, TX 77058, USA; 3 Chobanian and Avedisian School of Medicine, Boston University, Boston, MA 02118, USA

**Keywords:** vagus nerve stimulation, neuromodulation, mechanism, biomarker, drug-resistant epilepsy, epilepsy

## Abstract

Vagus Nerve Stimulation (VNS) therapy is a neuromodulation technique useful for the treatment of drug-resistant epilepsy and treatment-resistant depression. This article begins by reviewing the neuroanatomy and physiology of the vagus nerve. It then delves into recent advances in our understanding of VNS’s mechanism of action at different levels: how it affects different nerve fibers, how it affects neural pathways, and how it creates anti-inflammatory effects. This article then surveys research to adapt and optimize VNS, guided by an improved understanding of its mechanism of action and descriptions of its effects.

## 1. Introduction

Vagus Nerve Stimulation (VNS) Therapy uses an FDA-approved neuromodulation device for the treatment of drug-resistant epilepsy (DRE). It was initially approved for adults in 1997 and later for children aged 4 years and older with focal epilepsy. In Europe, VNS is approved for both focal and generalized epilepsy [1,2]. It is also used in the treatment of other conditions like depression [3]. For a detailed overview of patient selection, surgical procedures, and treatment outcomes, see Abdennadher et al. [4].

The mechanisms of action for VNS for epilepsy remain only partially understood. This article highlights emergent evidence about VNS mechanisms: modulating brain networks, balancing neurotransmitters, and impacting neuroinflammation. It then reviews potential biomarkers, treatment response predictors, and emerging approaches in VNS such as closed-loop systems.

## 2. Neuroanatomy and Physiology of the Vagus Nerve

The section reviews the neuroanatomy and physiology of the vagus nerve, as relevant to VNS. The vagus nerve is the main parasympathetic output of the autonomic nervous system. It arises from the medulla oblongata in the brainstem, where four nuclei support vagal function: two efferent and two afferent [5].

The efferent nuclei are the nucleus ambiguus and dorsal motor nucleus, providing motor output to the pharynx, larynx, soft palate, and tongue for swallowing and speech, and mediating parasympathetic control of the heart, lungs, esophagus, and gastrointestinal glands [5,6,7,8]. The nerve exits the jugular foramen and descends within the carotid sheath, posterior and between the internal jugular vein and the common carotid artery [9]. This mid-cervical segment of the left vagus nerve is the typical site for VNS electrode implantation (about 8 cm above the clavicle) in the invasive VNS procedure. The lead is tunneled subcutaneously to a pulse generator placed in the left upper chest or axillary region [10,11]. The left vagus nerve is used in VNS because it has fewer efferent cardiac fibers, reducing the risk of bradycardia or arrhythmia [11,12].

**Figure 1:**
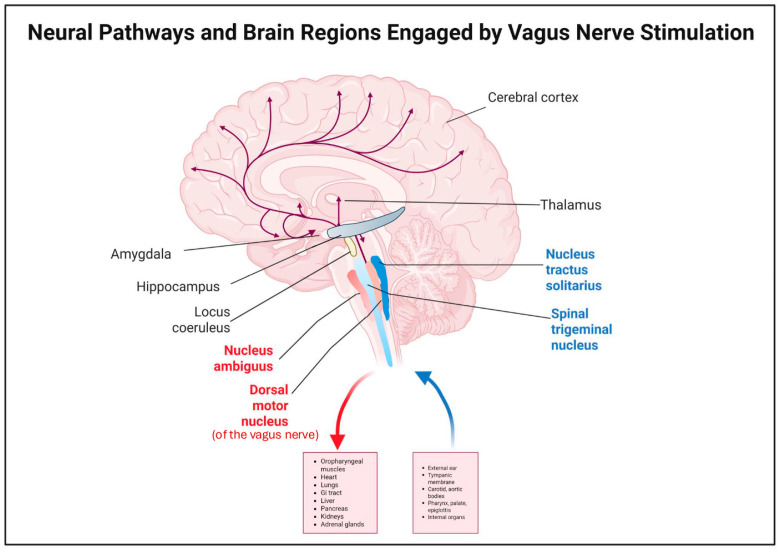
Illustration of the afferent and efferent pathways of the vagus nerve. Efferent fibers originate from the nucleus ambiguus and dorsal motor nucleus and extend to the pharynx, larynx, heart, lungs, and gastrointestinal tract [5,6,7,8]. Afferent fibers arise from sensory neuron cell bodies housed within the superior and inferior vagal ganglia and carry messages to the spinal trigeminal nucleus and nucleus tractus solitarius (NTS) [13]. Created by Created in BioRender. Cocoli, K. (2025) https://BioRender.com/283xy8u.

The afferent nuclei are the superior and inferior vagal ganglia, dealing with sensory input and making up 80% of the vagus nerve fibers [13]. Neurons from the superior ganglion send general somatic afferents to the spinal trigeminal nucleus, transmitting sensation from the outer ear and tympanic membrane, wherein the concha and tragus (in the ear) allow conduction in transcutaneous VNS [13]. Neurons from the inferior ganglion project to the nucleus tractus solitarius (NTS), which plays a central role in autonomic regulation. From there, they follow three major pathways: (1) to the autonomic motor system controlling visceral functions, such as breathing, heart rate, and blood pressure; (2) to the medullary reticular formation for respiratory reflexes; and (3) to the brain via connections with the hypothalamus, amygdala, limbic system, and cerebral cortex [6,7,14,15]. Of these, the NTS pathway to the cortex is especially important in epilepsy [8,16], as it enables broad cortical modulation through connections to noradrenergic and serotonergic central loci such as the locus coeruleus and raphe nuclei [17] (Figure 1).

## 3. Mechanisms of Action

This section reviews three proposed refinements to our understanding of VNS action mechanisms (Table 1).

### 3.1. Fiber-Specificity

The vagus nerve consists of myelinated A fibers (diameter > 10 μm), lightly myelinated B fibers, and unmyelinated C fibers (diameter < 0.5 μm), with C fibers making up over 80% of the total fiber count [18,19]. The fiber-specific hypothesis is that VNS acts by activating the A and B fibers, but not the C fibers.

**Figure 2:**
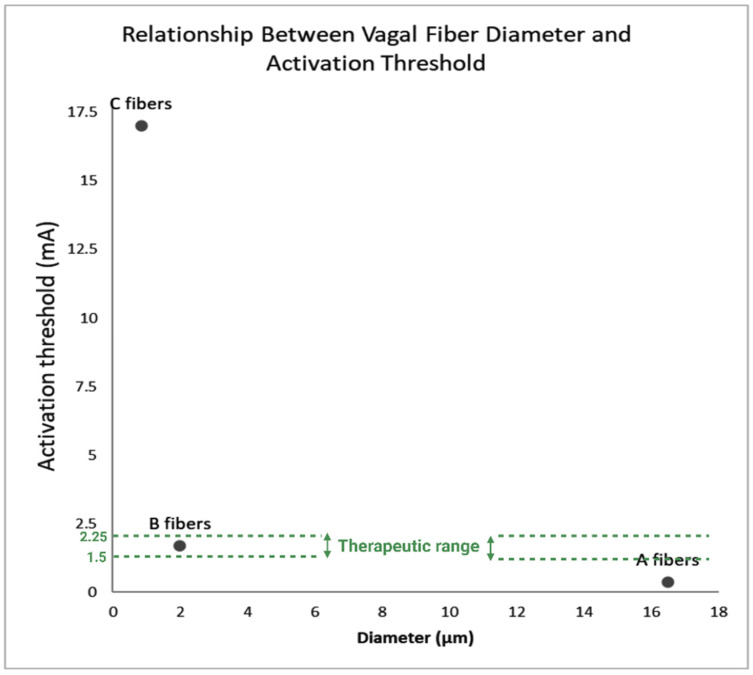
Relationship between vagal fiber diameter and activation threshold. The graph illustrates that larger, myelinated A fibers and lightly myelinated B fibers are activated within the therapeutic VNS range (1.5–2.25 mA), whereas small, unmyelinated C fibers (<0.5 μm) require much higher intensities (~17 mA) and remain largely inactive during clinical stimulation (1.5–2.25 mA) [19,24,25]. The increasing activation threshold with decreasing fiber diameter demonstrates why A and B fibers are considered the primary mediators of VNS effects. Created with BioRender by KC.

An earlier hypothesis was that C fibers were the primary therapeutic targets of VNS due to their high density and absence of myelin [20]. However, subsequent animal studies demonstrated that the activation threshold for C fibers is substantially higher than clinical VNS output (17.0 ± 2.6 mA versus 1.5–2.25 mA) [21,22,23], whereas A fibers respond at 0.37 ± 0.15 mA and B fibers at 1.6–1.8 mA, making A and B fibers more likely contributors to therapeutic effects [24,25] (Figure 2). The elevated C-fiber threshold is attributed to anatomical and physiological features such as thicker perineurium, larger fascicle diameters, and deeper location, which physically insulate fibers and reduce excitability [19,26,27]. Post-implantation factors, including electrode placement and fibrotic tissue growth, may further increase resistance and limit C-fiber recruitment [9]. This selective recruitment also explains why clinical VNS rarely elicits unpleasant sensations such as coughing, hoarseness, or apnea at standard output levels (1.5–2.25 mA) [28].

Animal studies support the predominant role of A and B fibers in mediating VNS effects. In experiments where the vagus below the stimulation site was ablated, or C fibers were selectively inactivated chemically, therapeutic outcomes persisted [29,30]. These findings suggest that myelinated fibers are sufficient to produce the anticonvulsant effects of VNS in animals. However, further research would need to test whether these results translate to humans, given differences in vagus nerve anatomy, fiber composition, and functional properties across species.

In humans, a few intraoperative studies have confirmed that A fibers respond at low stimulation intensities, while C fibers require much higher currents [31,32]. Early investigations using hook or forceps electrodes reported conduction velocities and activation thresholds that differed from those observed with clinical helical VNS devices [26,32]. This indicates that electrode design, stimulation geometry, and surgical setting strongly influence which fibers are recruited. Furthermore, recordings performed under anesthesia may not accurately represent fiber activation during chronic VNS in awake patients [18,28].

The discrepancies between animal studies, early human intraoperative data, and recent clinical recordings highlight important mechanistic gaps. Reliance on animal data alone is therefore limited, and systematic human studies using clinically relevant VNS systems are needed to clarify these uncertainties and optimize therapy.

### 3.2. Neural Pathways and Neurochemical Mechanisms

The therapeutic effect of VNS likely begins with activation of the NTS of the brainstem [16]. Vagus nerve afferent fibers end in the NTS, which is a central relay station for transmitting sensory information to other areas of the brain involved in seizure regulation, mood, arousal, and autonomic function. These other areas include the locus coeruleus (LC), parabrachial nucleus (PN), hypothalamus, thalamus, amygdala, and raphe nuclei, all of which either directly receive vagal afferent input or are highly interconnected with a bilateral hemispheric network [33,34,35]. VNS may act on this diffuse/bilateral neurostimulation network to regulate seizure thresholds and disrupt abnormal synchrony among epileptogenic circuits. In people with drug-resistant epilepsy, VNS stabilizes brain function by modulating cortical and limbic excitability through projection to the anterior cingulate cortex (ACC), prefrontal cortex, insula, and hippocampi [36,37]. Accumulating evidence from neuroimaging studies supports that VNS induces changes in the orbitofrontal cortex, brainstem, and limbic structures, highlighting a broad and bilateral network [38,39,40].

In addition to its effects on brain circuitry, VNS significantly influences the release of key neurotransmitters involved in seizure regulation. It increases noradrenaline release from the LC and serotonin from the dorsal raphe nucleus (DRN), both known to have inhibitory effects on cortical excitability [41,42]. Short- and long-term VNS is effective in elevating the discharge of LC neurons, thus leading to the accumulation of noradrenaline in regions like the prefrontal cortex, amygdala, and hippocampus [43]. Blocking hippocampal α (2)-receptors has been shown to reverse the seizure-suppressing effects of VNS, highlighting a strong positive correlation between the noradrenergic activity and anticonvulsive function [43]. Additionally, VNS is associated with increased GABAergic activity, which inhibits neurotransmission and reduces neuronal hyperexcitability, potentially enhancing hippocampal inhibition and contributing to seizure suppression [44,45,46]. These neuromodulatory effects suggest that VNS exerts its therapeutic benefits by restoring the balance between inhibitory and excitatory neurotransmission and thereby stabilizing neural circuits implicated in epilepsy.

### 3.3. Anti-Inflammatory and Cytokine Modulation Mechanisms

VNS produces anti-inflammatory effects that may contribute to seizure control. Preclinical studies have reported that VNS prevents neurotoxicity through the enhancement of kynurenine metabolites and normalization of total cortisol levels [47]. Through the modulation of the cholinergic anti-inflammatory pathway, VNS suppresses pro-inflammatory cytokines TNF-α, IL-1β, and IL-6 that are implicated in seizure generation and neuronal injury [48,49,50]. At the same time, VNS-treated patients show higher levels of anti-inflammatory cytokines, including TGF-β and IL-10, indicating a neuroprotective and immunomodulatory response [50,51]. Such additive effects might enhance increased neuroprotection and allow for long-term seizure control.

At the cellular level, VNS has been demonstrated to activate α7nAChR receptors in microglia, causing reduced inflammation and PI3K/Akt signal pathway activation, inhibiting apoptosis [50]. While this process is clearly defined in ischemic injury models [52], its precise function in epilepsy is not yet fully established.

Emerging evidence shows that epilepsy is associated with structural and functional blood–brain barrier (BBB) alterations that contribute to neuroinflammation and seizures. Preclinical models emphasize the microbiota–gut–brain axis and the vagus nerve as key regulators of systemic and neural inflammation [53], effects relevant not only to epilepsy but also to other neurological disorders. Activation of α7nAChR in splenic macrophages reduces cytokine release, enhances BBB integrity, and may protect against seizures, primarily via peripheral vagal–splenic signaling rather than direct central nervous system actions [54]. Ongoing research investigates how central nervous system inflammation and microglial activation mediate the therapeutic effects of VNS [55].

## 4. Measuring and Describing Treatment Response

### 4.1. Functional Connectivity as a Descriptor of Treatment Response

Functional connectivity is the correlation of activity between different brain regions, often measured by scalp electroencephalogram (EEG), intracranial EEG (iEEG), or functional neuroimaging [56]. Epilepsy involves excessive synchronization—often between regions—of a group of neurons, leading to seizure [57,58]. Epilepsy also sometimes involves the disruption of healthy communication—and connectivity—between regions. Functional connectivity can therefore help describe epileptic networks that would benefit from VNS by disrupting this excessive synchronization. Studies also suggest that VNS improves communication within and between thalamic nuclei, particularly at the centromedian and the anterior nucleus of the thalamus [59].

**Figure 3:**
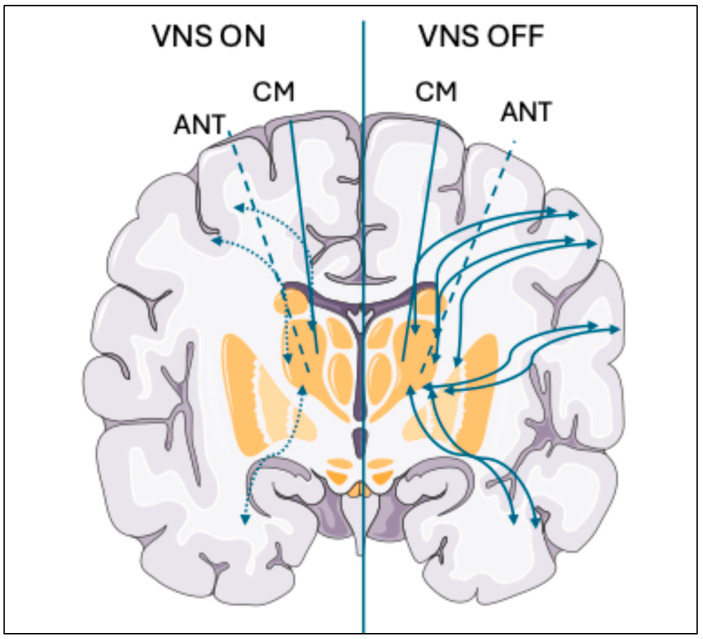
EEG connectivity changes during VNS ON vs. VNS OFF phases. Schematic representation shows lower thalamo-cortical connectivity during the ON phase of VNS (stimulation phase: left panel) compared to the OFF phase (no stimulation phase: right panel). This reduction reflects neural desynchronization, likely contributing to lower risk for seizure [59,60]. Illustration adapted from Servier Medical Arts at smart.servier.com.

Patients responding to VNS show a significant global reduction in EEG connectivity, especially in the gamma frequency band, during the ON phases (when the device is sending stimulation) compared to the OFF phases (i.e., pause) [60] (Figure 3). Reduced network connectivity implies reduced synchrony and, therefore, possibly reduced likelihood of seizure [61]. Preclinical (animal) research has explored the electrophysiological basis for such connectivity changes, revealing that rapid-cycling VNS (7 s On, 18 s Off) suppresses hippocampal activity in rats more effectively than standard cycling (30 s On, 300 s Off), even at lower current (0.25 mA versus 1.0 mA), with fewer side effects [62]. This study also showed stimulation-induced reduced theta-gamma phase–amplitude coupling (PAC)—possibly inhibiting seizure propagation by limiting the recruitment of neurons from other brain regions [63].

Additionally, baseline thalamic resting-state functional connectivity (rsFC) may predict seizure reduction after microburst VNS (μVNS), notably in focal-onset epilepsy. This study showed that stronger baseline connectivity between the thalamus and parietal regions was associated with seizure reduction [64].

### 4.2. Imaging-Based Descriptors of Treatment Response

Brain imaging in humans and animals has provided critical information on the mechanism of VNS. Early experiments identified VNS-induced activity within autonomic and affective processing regions, such as the amygdala, thalamus, hypothalamus, and brainstem [65]. Human PET scans later confirmed increased blood flow to these areas as well as decreased activity in the hippocampus and amygdala—patterns congruent with the seizure-suppressant effect of VNS. Henry et al. [65] used [O-15] water and PET perfusion imaging to show thalamic and temporal cortical activation, regions also named as being accountable for functional connectivity changes revealed through EEG and MEG.

While these acute responses are well-characterized, long-term VNS therapy may lead to adaptive network changes that are more subtle and distributed, contributing to long-term therapeutic effects. Notably, the spatial extent and direction of both acute and chronic responses vary considerably among individuals. Recent observations using iEEG recordings have identified a nonlinear relationship between VNS parameters and the resulting brain responses. Scholars reported inter-individual variability with increased or decreased connections between different brain regions, and decreased functional connectivity in VNS responders [66]. These findings highlight the complexity of neuromodulation and suggest that effective VNS therapy will have to be more personalized and adaptive in its programming approaches, rather than simply raising stimulation amplitude or rate [31].

## 5. Advances in VNS Methods

This section discusses adaptations and improvements to VNS, including electrode placement, syncing stimulation to physiological markers, and stimulation parameter optimization.

### 5.1. Current Clinical Outcomes

Despite early skepticism, growing clinical evidence supports VNS as a long-term treatment for drug-resistant epilepsy. A recent retrospective study showed 75% of patients with at least 5 years of follow-up achieved at least 50% seizure reduction [67]. This aligns with prior findings of improved intelligence quotient, quality of life, and decreased psychiatric and behavioral problems [68], underscoring VNS’s multidimensional benefits [3].

Another study reported 65% of patients experiencing improvements in seizure frequency, duration, and severity, with hoarseness as the most common side effect (70%). Only 12% found side effects intolerable. Early response within the first month was a strong predictor of long-term benefit, and devices can be removed if ineffective [69]. For more details, see our previous publication on VNS methodology and clinical outcomes [4].

### 5.2. Technological Innovation and Parameter Optimization

New technologies and treatment strategies are challenging conventional VNS practices, including electrode placement [70]. Left-sided placement is typical, with the understanding that this helps avoid cardiac risks such as asystole and bradycardia associated with right-sided placement. In a study of 38 patients with right-sided placement, severe cardiac complications did not occur in the majority of patients. Although these results suggest that right-sided placement may be safe and effective (important if it is the only viable option for a particular patient), the small sample size warrants caution. Potential selection bias and the need for careful safety monitoring should be emphasized when interpreting these findings [70].

Microburst VNS (μVNS) is a stimulation approach designed to enhance the efficacy of vagus nerve stimulation while minimizing side effects. In contrast to standard VNS, which delivers constant or cyclical stimulation, μVNS consists of high-frequency pulse trains (100–350 Hz in 50 Hz increments), consisting of 4–7 pulses per burst, followed by longer off periods [71] (Table 2). Preclinical rodent research has indicated that μVNS, unlike traditional VNS, enhances synchronization of locus coeruleus neuron firing. This effect is believed to be more efficient because it mimics the intrinsic phasic firing pattern of the locus coeruleus [72]. In a recent clinical trial, μVNS reduced seizures in drug-resistant epilepsy, with 67% of focal and 80% of generalized seizures showing more than 50% improvement at 12 months. MRI analysis showed that μVNS changed the connectivity of the thalamic brain patterns, significantly in focal epilepsy, with higher initial connectivity predicting better response. These findings suggest that μVNS may target the thalamic network level to produce its effects [64]. While these results are promising, more studies are needed to fully understand how μVNS works and which patients will benefit the most.

Transcutaneous VNS (t-VNS) is a newer, noninvasive alternative to traditional VNS that offers patients a gentler treatment option. A few meta-analyses revealed that t-VNS significantly reduced seizure frequency in patients with refractory epilepsy, particularly over longer durations [73]. Improvements in quality of life were also reported, with only mild and temporary side effects—primarily headaches, which were reported even less frequently than in control groups [73,74,75,76,77,78].

Studies have explored how patient-related factors may impact the outcome of t-VNS. One study found that patients with elevated initial seizure frequencies demonstrated a significantly stronger therapeutic effect. However, no meaningful correlations were identified between seizure reduction and age, quantity of AEDs, MRI or EEG results, initial stimulation strength, or type of seizure [78]. This indicates that t-VNS could be beneficial for a variety of patient profiles, supporting its potential in broader clinical applications. However, standardization of stimulation protocols and electrode placement across studies is needed to improve comparability and guide clinical practice.

Other neuromodulation therapies have shown promising results. These include transcranial direct current stimulation (tDCS), like t-VNS, used in drug-resistant epilepsy [79]. Deep brain stimulation and responsive neurostimulation are invasive neuromodulation methods used individually or in combination with VNS and approved for drug-resistant epilepsy. They can be selected based on specific indications or patient preference [80,81,82].

A nationwide study comparing VNS and Laser Interstitial Thermal Therapy (LITT) revealed that both these treatment options continue to be significantly underutilized in adult patients with refractory epilepsy (only 0.34% received LITT and 0.66% received VNS) [83]. Both treatment options displayed similar complication rates, hospital stay lengths, and discharge outcomes, although LITT was associated with slightly lower hospitalization costs.

### 5.3. Future Research Directions and Challenges

The focus of VNS research is increasingly on individualized, adaptive, and integrated neuromodulation systems. A “one-size-fits-all” programming paradigm is likely to create suboptimal efficacy or potential side effects. Current attempts are focused on combining real-time biomarkers, including heart rate variability (HRV), ictal tachycardia, electroencephalogram (EEG) pattern, and pupil dilation response (PDR), with closed-loop or artificial intelligence (AI)-guided VNS devices that automatically modulate stimulation parameters to patient-specific neurodynamics [83,84,85,86].

Researchers are developing ways to optimize the administration of VNS, in large part focused on physiological markers and patient factors. Since most seizures are preceded by ictal tachycardia [87], this marker has been used to develop closed-loop VNS systems that deliver stimulation in real time to reduce or abort seizures [88]. Pupil dilation response (PDR) is another biomarker that could help optimize stimulation levels and avoid overstimulation [89,90]: PDR shows an inverted U-shaped response, with dilation peaking at 2–3 s after stimulation before declining. However, the reliability of PDR requires validation in larger studies.

Besides electrophysiological markers, novel research is also unraveling molecular biomarkers that can further refine individualized treatment. A study investigating the effects of long-term VNS on fatty acids and lipid bioactive metabolites in humans found that stimulation to therapeutic levels increased SIRT1 and PPARα gene expression—two critical regulators of lipid and energy metabolism that can lower saturated fatty acid content in erythrocytes [46]. These findings suggest that VNS may improve neuronal function by modulating systemic energy metabolism and offer a new molecular mechanism of seizure termination in drug-resistant epilepsy [46].

The interaction of VNS with other neuromodulation methods could further optimize treatment. Concurrent application of VNS with deep brain stimulation (DBS) or responsive neurostimulation (RNS) provides potential synergistic benefits, especially for multifocal or generalized epilepsies with less sensitivity to one-site stimulation [91,92]. In the same vein, blending invasive VNS with noninvasive measures like transcutaneous auricular VNS (t-VNS), transcranial direct current stimulation (tDCS), or transcranial magnetic stimulation (TMS) might maximize network-level control and decrease invasiveness [92]. Direct comparison or combination of these modalities in forthcoming clinical trials is warranted to identify the most effective multimodal regimens. The chronic ambulatory electrocorticographic recording offers a unique opportunity to shape treatment and epilepsy knowledge. With continued electrocorticographic activity recording and monitoring of aberrant events in the long term, the system offers personalized detection and stimulation parameters that are tailored to real-time brain activity, in contrast to short-term inpatient EMU monitoring [91]. Ambulatory data would also assist in earlier and more precise antiepileptic drug titration [89]. In addition, chronic intracranial EEG can identify temporal lobe epilepsy patients who may benefit from a resection [92]. Subsequent studies using machine learning and deep learning techniques can potentially further optimize detection and stimulation strategies, both on a subject-by-subject [93].

In addition, multimodal neuroimaging and connectomics will likely have a role to play in rendering personalized VNS treatment. A patient-brain connectivity map may have the potential to predict responders and guide electrode placement or parameter optimization. Large-scale, long-term studies integrating neuroimaging, electrophysiology, and immunological biomarkers will be needed to gain complete mechanistic insight into the heterogeneity of VNS and translate such knowledge toward precision neuromodulation [94].

## 6. Conclusions

VNS is a helpful therapy for drug-resistant epilepsy that offers benefits beyond seizure control, including improved cognitive, affective, and physiological functioning. While its exact mechanisms remain incompletely understood, recent research has identified the contributions of brain network modulation, neurotransmitter balance, and anti-inflammatory effects. Innovations in functional connectivity studies, imaging biomarkers, and adaptive stimulation technology are enhancing our ability to describe treatment response and optimize outcomes. While the field progresses toward more personalized and noninvasive alternatives, VNS remains a valuable neuromodulator intervention that can be tailored to individual patients’ needs. Future research using multi-omics, advanced imaging, or neuroinflammatory biomarkers may lead to the discovery of novel treatment response biomarkers.

Despite promising advances in VNS therapy, there are several limitations to be acknowledged. First, the precise mechanisms of VNS action remain unknown. Second, much of the current evidence relies on preclinical animal studies and small clinical cohorts, which may limit generalizability and introduce potential selection bias. Finally, there are no consistent stimulation parameters—frequency, pulse width, and electrode location—across studies to enable results comparison and the optimization of treatment strategies. Addressing these limitations will be necessary to guide future studies and improve individualized VNS therapy.

## Figures and Tables

**Table 1:** Proposed VNS mechanisms.

Mechanism	Description
Fiber-Specificity	VNS primarily activates myelinated A and B fibers, not unmyelinated C fibers, due to lower stimulation thresholds. These fibers mediate therapeutic effects without causing unpleasant sensations. [9,18,19,20,21,22,23,24,25,26,27,28,29,30,31,32]
Neural Pathway Effects	Vagus afferents project to the NTS and connected regions (LC, PN, hypothalamus, thalamus, amygdala, raphe nuclei). VNS increases noradrenaline, serotonin, and GABA activity to stabilize brain function and control seizures. [16,33,34,35,36,37,38,39,40,41,42,43,44,45]
Anti-Inflammatory Effects	VNS activates the cholinergic anti-inflammatory pathway, reduces pro-inflammatory cytokines (TNF-α, IL-1β, IL-6), increases anti-inflammatory cytokines (IL-10, TGF-β), and enhances microglial and macrophage-mediated neuroprotection. [46,47,48,49,50,51,52,53,54]

**Table 2:** Comparison of Standard VNS and Microburst VNS (μVNS) Features and Clinical Outcomes.

Feature	Standard VNS	Microburst VNS (μVNS)
Frequency	Typically 20–30 Hz	100–350 Hz (bursts)
Pulse pattern	Continuous or cyclical	4–7 pulses per burst with long off periods
Mechanism	Broad VNS activation	Enhances locus coeruleus synchronization; targets thalamic network
Clinical efficacy	50–60% patients show >50% seizure reduction	67–80% patients show >50% seizure reduction in recent trials
Side effects	Voice alteration, cough, throat discomfort	Potentially reduced due to a brief burst pattern

## Data Availability

Not applicable.
